# Discovering the allure of forests: Exploring adolescent queries in nature-rich environments

**DOI:** 10.1371/journal.pone.0312955

**Published:** 2025-01-16

**Authors:** A. M. Arroz, R. Gabriel, A. R. Silva, F. Piasentin, I. R. Amorim, A. Picanço, S. Matos

**Affiliations:** 1 cE3c/ABG—Centre for Ecology, Evolution and Environmental Changes, Azorean Biodiversity Group, CHANGE–Global Change and Sustainability Institute, University of the Azores, Angra do Heroísmo, Azores, Portugal; 2 School of Social Sciences and Humanities, University of the Azores, Ponta Delgada, Portugal; 3 School of Agricultural Sciences and Environment, University of the Azores, Angra do Heroísmo, Portugal; 4 Center of Agrarian, Environmental and Biological Sciences, Federal University of Recôncavo of Bahia, Cruz das Almas, Bahia, Brazil; 5 Interactive Technologies Institute (ITI/LARSyS), Polo Científico e Tecnológico da Madeira, Funchal, Portugal; 6 Faculty of Design and Art, Free University Bozen-Bolzano, Bolzano, Italy; National Open University, TAIWAN

## Abstract

This study explores adolescents’ inherent curiosity about nature through the production of self-generated questions during a field visit to a nature-rich environment, followed by descriptive-interpretative analysis using focus groups. Utilizing cultural probes and content-free question tokens, we collected 164 valid questions produced by 36 adolescents during the field session. Biotic elements, like species, turned out to be more intriguing than abiotic elements, originating 89.6% of the questions. The predominant topics were related to species adaptation, extinction, dispersion, and diversity, with younger adolescents showing a notable interest in nature conservation, while older adolescents highlighted biodiversity dynamics. These findings were corroborated by the ranking of the TOP-5 most interesting questions, where biodiversity dynamics, nature conservation and plant physiology occupied the same relative positions. Our results indicate that in a nature-rich environment and through an inquiry-based approach, adolescents were encouraged to express curiosity about nature. This approach could be a valuable educational strategy to enhance their connection to nature, promote conservation responsibility, and benefit the environment.

## Introduction

The need to deepen our understanding of nature as a ‘space of childhood’ [1] together with the decrease of youth’s interest in nature [2–5] arises from a confluence of epistemic and pragmatic factors. On one hand, since we recognize children and young people as active sense-makers with full rights [6], it becomes essential to explore their relationship with the natural world. However, despite acknowledging their distinct perspectives on nature [1, 7, 8], there remains a shortage of knowledge regarding how child and youth cultures relate to the natural world [1, 9–11]. On the other hand, over the past decades, children and adolescents have diminishing direct experiences with nature [12–15], now replaced by digital communication technologies [13, 16, 17]. The detachment from nature during childhood and adolescence [18] has led to a decline in contact, connection, engagement, and interest in nature [4, 19–23]. This diminishing relationship has potential implications for both individual well-being [24] and environmental sustainability (e.g. [25]).

Understanding nature’s place in contemporary experiences of childhood and adolescence is essential to reverse these trends. Several studies have attempted to explore the theme by asking: (i) how children and adolescents conceptualize nature [10, 11, 19, 26–31] and (ii) how they connect and experience nature [6, 11, 19, 26, 28, 31–34]. A summary of empirical studies main findings may be seen in S1 Table.

To date, research on these matters has primarily relied on qualitative methodologies, focusing on children and young adolescents in developed countries. These studies have uncovered a wide spectrum of viewpoints, ranging from detachment from nature to a dedicated commitment to environmental conservation. Consequently, it is of utmost importance to pinpoint the factors that contribute to this diversity, recognizing that they can differ based on various variables such as individuals’ group, rural/urban setting, age, and specific locations being examined (S1 Table).

Interestingly, a number of studies show that adolescents are less environmentally friendly and less involved in natural issues than both children and young adults, a phenomenon sometimes denominated time-out [20, 35], or dip [23]. This phenomenon was documented on pro-environmental attitudes, behaviors and preference for natural settings [20], concerns [4], nature connectedness, emotional affinity and prescriptiveness of moral judgment [36], and sustainability consciousness [23]. This detachment from nature may stem from specific tasks of adolescence, which are more self-centered than context-oriented [36, 37]. A more recent global synthesis by Soga & Gaston [38] highlights the widespread occurrence of the shifting baseline syndrome, where generational detachment from nature is increasingly prevalent, especially during adolescence.

However, over the past three decades, the research landscape concerning children and adolescents’ perceptions and experiences of nature has been notably sparse. A critical gap exists in the investigation of the specific aspects of nature that captivate adolescents. Conducting comprehensive studies in this area can yield valuable insights into the factors that ignite adolescents’ curiosity about nature. By addressing this research gap, we can contribute to a better understanding of the relationship between young individuals and nature and potentially encourage them to reconnect/engage with the natural world.

Examining the type of questions individuals formulate about a specific topic can be a valuable way to identify their curiosities and interests [39–41]. Questions often reveal one’s thinking process and their desire to expand knowledge and understanding of new ideas [42]. For instance, a study on Israeli children’s interest in scientific issues involved analyzing their self-generated questions, which were predominantly factual, followed by explanatory, methodological, and open-ended questions [39]. The motivations behind the questions varied depending on participants’ age groups, with younger children asking fewer applicative questions and showing more curiosity about the expected dimensions of biology and questions related to personal use, compared to adolescents and adults [43].

The active information seeking process lies at the interface between curiosity and interest–a domain yet to achieve conceptual clarity and consensus (cf. review in [44]). In general, interest can be understood as the desire to engage with an object or to acquire knowledge about a specific topic [45, 46], involving emotional, value-related, and cognitive components [47]. There are two types of interest: *individual interest*, which is a personality trait, and *situational interest*, which is a psychological state. On the other hand, curiosity is a multidimensional concept related to information-seeking activities. It can be classified into two types: a feeling of deprivation and a feeling of interest in learning something new [48]. Various reviews have explored the relationship between curiosity and interest, considering the possibility of using these terms interchangeably or seeing them as distinct processes [44, 49].

While self-generated questions have been utilized in several studies to identify students’ curiosities and interests in subjects like informal science topics [50], scientific issues [51], and biology [43, 52], none, to the best of our knowledge, have specifically focused on nature, in the broadest sense. Therefore, this study aims to investigate adolescents’ questions regarding nature, considering the context of the natural environments of the Azores, a biodiversity-rich archipelago. These questions might represent different phenomena within the participants’ conceptual ecologies, arising from situational interests or curiosities, momentarily triggered by the tasks conducted at each station of the trail. To guide our investigation, we formulated the following research questions:

What insights can we gain from adolescents’ self-generated questions about their curiosities regarding nature? Which objects and topics appear most prominently? What knowledge do they seek to acquire through their questions?

Are there any discernible differences between the questions posed by younger and older adolescents?

What factors make certain questions more relevant to adolescents compared to others?

This study aims to offer insights to educators and conservationists by examining adolescents’ self-generated questions about nature. Through the analysis of their curiosities and interests, we seek to help design activities and place-based environmental education interventions tailored to their needs. Furthermore, understanding what appeals to adolescents may provide a good starting point to foster engagement and reconnection with nature, contributing to their wellbeing and physical and mental development.

## Methodology

In this study, adolescents participated in an activity which involved exploring diverse natural environments through a trail hike, on the 1st and 7th of December 2019. Written informed consent was obtained from their parents or guardians. During the hike, adolescents engaged in various tasks along stations throughout the trail using the cultural probes approach. One of the tasks required in each station was to produce questions that expressed their curiosity about nature [53]. This research focuses on analyzing and interpreting the questions adolescents formulated during the field activity and on the views they expressed regarding the degree of curiosity evoked by the questions, made during a post-activity session at the University of the Azores in Angra do Heroísmo, on the 25th of January 2020. The study received approval from the Ethics Committee of the University of the Azores (Portugal) under Declaration number 45/2019.

### Study context and participants

The study took place on Terceira Island, in the Azores archipelago (Portugal), located in the North Atlantic Ocean. Covering an area of 400 km^2^ and with a population of 53,311 people [54], Terceira is home to about 6,000 adolescents aged between 10 and 18 [55]. The Nature Park of Terceira comprises 20 protected areas, including the ‘Nature Reserve of Serra de Santa Bárbara and Mistérios Negros’. This reserve is known for its diverse Azorean natural vegetation and animal life, as well as its small bodies of water and volcanic domes formed during an eruption in 1761 [56]. The circular 5 km ’Mistérios Negros’ trail stands out for its biological and geological diversity and its accessibility, making it an ideal location to investigate how adolescents interact with and experience the natural environment.

We enrolled a total of 36 adolescents from two local scout groups, 19 girls and 17 boys, with little to no knowledge of the visited space (Matos et al., 2022). Among them, 23 were ‘explorers’ (10 to 14 years), and the remaining 13 were ‘pioneers’ (15 to 18 years). Throughout the study, we refer to them as ’younger adolescents’ and ’older adolescents,’ respectively. Participants’ free evocations about the ’natural forests of the Azores,’ collected just before the hike, characterized these forests by their substance (e.g. ‘nature’), biotic components (e.g. ‘trees’, ‘animals’), and the sensations they allow (e.g. ‘fresh air’, ‘peace’).

### Information seeking, collection methods and procedure

The activity in the ‘Mistérios Negros’ trail was oriented by a *place-based* approach incorporating potential triggers for interest including, besides the trail, *hands-on experiences*, *novelty* and *group work* [57]. All participants started at the trail’s beginning (station 1) and were then grouped into pairs or triads. Along the first two kilometers of the trail, there were twelve additional stations, each with a unique task (Table 1). Six groups of younger adolescents and five groups of older adolescents explored different sets of stations (2–7 and 8–13, respectively), but the stations had similar structure and purpose [53, 58, 59]. Tasks were designed to cater to multiple intelligences and revolved around various natural elements, aiming to enhance participants’ awareness and engagement with the forest. After each task, the groups were encouraged to produce two or more questions using content-free question tokens, following King’s approach [60]. Participants could choose from nine tokens, including connection questions (e.g., ’Explain how. . .’) and comprehension questions (e.g., ’Why is. . . important?’). Instructions on how to use the tokens were provided on a separate sheet, where adolescents also wrote their self-formulated questions. At the conclusion of the activity, participants engaged in a final closure task (station 14).

**Table 1:** Name of the 14 stations where adolescents engaged in nature-related tasks, before producing self-generated questions.

Younger adolescents	Older adolescents
Station 1. Starting activity	
Station 2. The mystery of water	Station 8. **Touch and feel**
Station 3. The smells of the forest	Station 9. **Manager for 10 min**
Station 4. Help the forest	Station 10. **Where are the animals?**
Station 5. Create your nature reserve	Station 11. **Hear the Forest**
Station 6. Who lives here?	Station 12. **Lime-Green-Bud-Green**
Station 7. Where am I?	Station 13**. Travel in time**
Station 14. Final activity	

*Note*. See activities details and instructions in other publications [53, 58].

We organized a post-trail activity a month and a half later, in a classroom environment, at the University of the Azores. Here, we collected data concerning the relevance of the questions, designing the session to allow participants to (1) rank the questions previously formulated in the field according to their will of seeing them answered by experts and (2) justify their choices for the TOP-5 most interesting questions. Of the 36 initial participants, 33 participated in the post-trail activity. We divided them into 11 groups of three, with younger and older adolescents in distinct groups.

**Fig 1:**
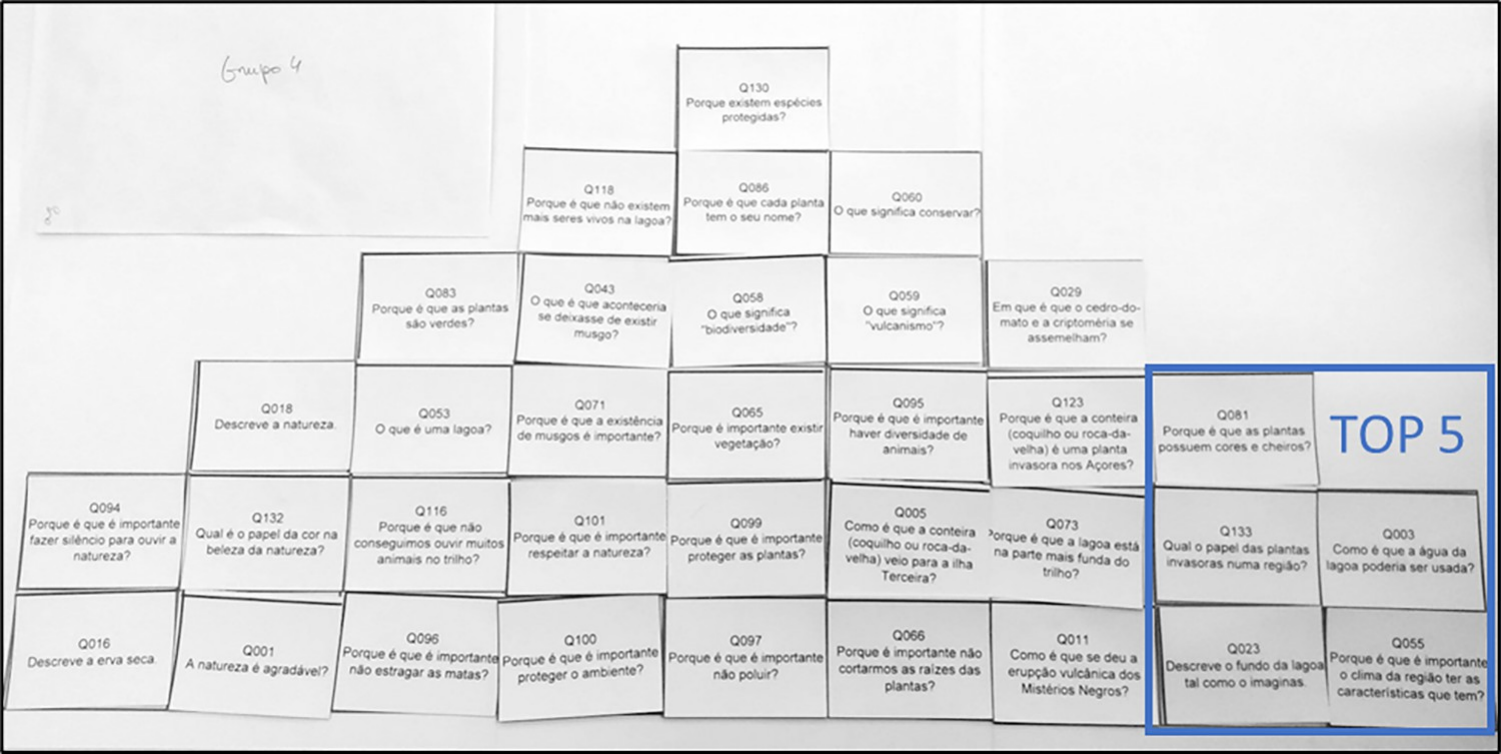
Example of a Q-sort pyramid with 34 questions ranked according to participants’ interest in seeing them answered by experts.

To design a meaningful activity for participants to rank the questions, we drew inspiration from the Q-sort methodology [61], aiming to encourage a participatory selection process. From the 164 valid questions collected during the intervention, we identified 137 distinct questions. These were printed on paper cards and divided into four groups of 34, later ranked by 11 groups of three participants. Each triad ranked the questions according to relevance in a nine-column paper pyramid with 34 blank spaces, starting at the bottom right (the most interesting) and moving left (the least interesting) (Fig 1).

Following the ranking process, participants identified the TOP-5 most interesting questions within their group and provided written justifications for their selections, resulting in a total of 55 questions.

### Data analysis

Our analysis of the questions followed an inductive process to identify common themes. Three themes emerged from the data: the material **objects** on which the questions focus; what participants **aim to know** about these objects; and the **topics** or frameworks in which questions are embedded. For instance, in the question ‘What will happen if we don’t preserve plants?’, the object refers to plant species, the aim is to know the impacts of action/inaction, and the topic is nature conservation.

We transcribed and segmented the data collected during the field and post-trail activities into a Microsoft Excel file. To identify common themes and patterns of meaning in the questions and justifications formulated by participants, we conducted a thematic analysis [62]. Our approach was inductive, allowing the categories to emerge directly from the data, using both explicit (semantic) and interpretative (latent) methods to identify participants’ curiosities about nature. The system of categorization was developed *a posteriori* and was continually refined until it achieved full coverage of the units of meaning present in the data. We provide further details on the operationalization of the categories in the Results section.

Two team members (AMA; ARS), each with a different academic background (Environment Psychology; Nature Conservation and Management), collaboratively developed the coding process through iterative reformulation of the categories, by negotiating meanings, until all were clarified and defined in operational terms. Additionally, four external judges conducted an intercoder reliability test using Cohen’s Kappa coefficient. The test involved a random sample of 42 units of meaning out of the 237 that had emerged from the data. Initially, the concordance coefficients ranged from 0.49 to 0.85, indicating some disagreements. These discrepancies were addressed by redefinition and refinement of some categories, resulting in near-perfect final coefficients ranging from 0.97 to 1.00.

We conducted a comparison of younger and older adolescents’ categories for each theme to examine potential age group differences. Additionally, we compared the TOP-5 questions with the entire set of questions (TOTAL) to identify the most interesting objects, aims, and topics to adolescents, and to understand why these specific types of questions are relevant to them. To explore differences between age groups and the groups of questions, we employed Pearson’s Chi-square tests (χ2) with a 95% confidence level; whenever more than 20% of expected frequencies were below five, we used the likelihood ratio value instead of Pearson’s Chi-square value (χ2). All tests were performed on SPSS Statistics Version 27 [63].

All the examples of questions formulated by adolescents used in this article were translated from the original Portuguese version to English.

## Results

Out of the total of 168 questions formulated by adolescents, four could not be categorized (https://doi.org/10.1371/journal.pone.0262853.s001). Among the 164 valid questions, 137 were distinct, indicating a high level of curiosity in nature. While we identified nine different types of *objects* that participants’ questions focused on and eight different *aims*, and the number of *topics* that appealed to the participants was even higher, reaching 14.

### RQ1: Which objects are focused on participants’ questions?

Table 2 illustrates the nine object types or entities of the physical reality that the questions focus on.

**Table 2:** Objects in focus and respective contents and examples of adolescents’ questions about nature (N_Participants_ = 36; N_Questions_ = 164; n, number of formulated questions in each object category).

Objects	Content	Examples
*Species* (n = 93)	Include questions related to plants, animals, and biodiversity; habitat type, encompassing questions focusing on lagoons, forests, and pastures	‘Why is peat moss important?’; ‘Why is the number of birds we hear so low?’; ‘Why is biodiversity important?’
*Habitats* (n = 31)	Encompass questions focusing on lagoons, forests, and pastures	‘Why is the lagoon in the deepest part of the trail?’; ‘What can I do to help the forest?’; ‘How do nature and farmland differ?’
*Nature* (n = 23)	Center on different types of protected areas, nature conservation, and nature in general	‘What is a protected area?’; ‘What does it mean to conserve?’; ‘Why is nature pleasant?’
*Nature trails* (n = 5)	Refer specifically to questions about the ’Mistérios Negros’ trail or nature trails in general	‘Why is the trail of Mistérios Negros rich in plant species?’; ‘Why is it important to walk on trails?’
*Environment* (n = 5)	Include questions focusing on broader contexts that are not explicitly natural	‘How can we preserve the environment?’
*Volcanic eruptions* (n = 4)	Involve questions related to volcanism	‘How did the volcanic eruption of Mistérios Negros take place?’; ‘What does *volcanism* mean?’
*Climate* (n = 1)	Pertain to the characteristics of the regional climate	‘Why is it important for the region’s climate to have the characteristics it has?’
*Recycling* (n = 1)	Encompass very specific questions preventing their inclusion in other object types found in the data	‘Why is recycling important?’
*Scent* (n = 1)		‘Why are some smells stronger than others?’

**Fig 2:**
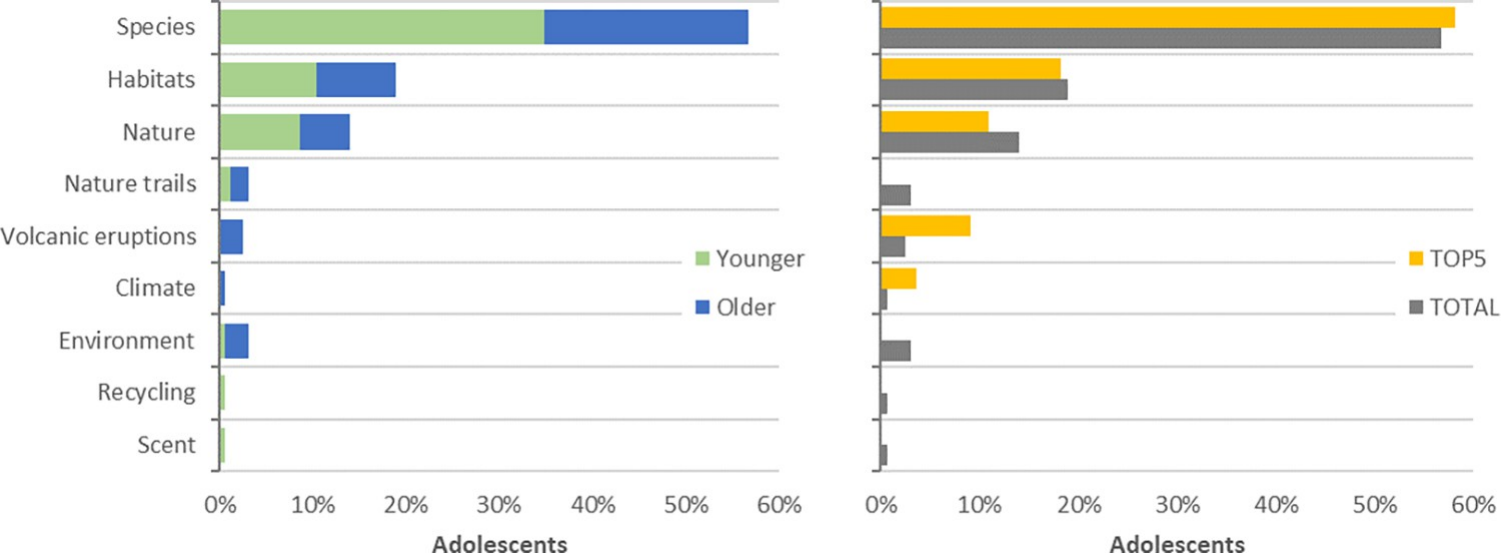
Objects in focus on participants’ questions about nature (Younger adolescents: N_Younger_ = 23; Groups_Younger_ = 8; Questions_Younger_ = 93; Older adolescents: N_Older_ = 13; Groups_Older_ = 6; Questions_Older_ = 71; Questions: N_Total_ = 164; N_TOP5_ = 55): (a) Comparison between adolescents’ age groups; (b) Comparison between the TOP-5 most interesting and the total valid questions.

The majority of adolescents’ questions were focused on *species* (57%), followed by *habitats* (19%) and *nature* (14%). No significant statistical differences were found among the distributions of types of objects in focus between younger and older adolescents (Fig 2A; χ2(8) = 15.042; *p* = .058), although some types appear as the focus of only one of the age groups (e.g., *volcanic eruptions*).

Furthermore, upon comparing the total valid questions with the TOP-5 most interesting questions, we observed no significant statistical differences in the distribution of the two groups of questions (Fig 2B); χ2(8) = 13.335; *p* = .101). *Species* remains the dominant type of object among the TOP-5 questions (58%; n = 32), followed by *habitats* (18%; n = 10) and *nature* (11%; n = 6)–a distribution very similar to the total group of questions.

Notably, all questions focused on *volcanic eruptions* were included in the TOP-5 group, while questions related to na*ture trails* and the *environment* didn’t make the cut.

### RQ2: What do participants aim to know with their questions?

**Fig 3:**
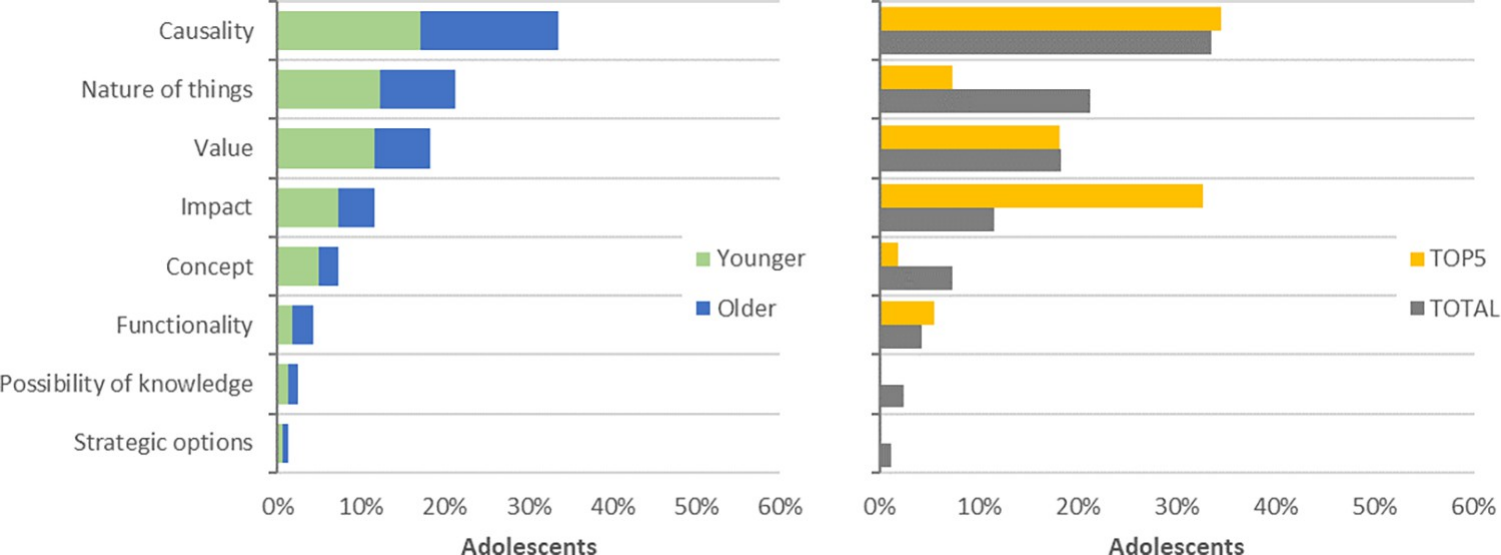
*Aims of the participants’ questions about nature* (Younger adolescents: N_Younger_ = 23; Questions_Younger_ = 93; Older adolescents: N_Older_ = 13; Questions_Older_ = 71; Questions: N_Total_ = 164; N_TOP5_ = 55): (a) Comparison between adolescents’ age groups; (b) Comparison between the TOP-5 most interesting and the total valid questions.

During the categorization of questions based on the participants’ aims in acquiring knowledge about the mentioned objects, we identified eight distinct aims (Table 3; Fig 3).

**Table 3:** Aims of the questions, contents and examples of adolescents’ questions about nature (N_Participant_ = 36; N_Questions_ = 164; n, number of formulated questions in each category).

Aims	Contents	Examples
*Causality* (n = 55)	Questions seeking an explanation of events connecting two or more phenomena, where one acts as the cause and the other as the effect	‘How did invasive plants get here?’; ‘Explain why it smells like wood on this trail.’
*Nature of things* (n = 35)	Questions inquiring about the essence of a phenomenon and/or the description of something, exploring its ontological dimension, including its substance and relationship with its name	‘Describe the beauty of the lagoon.’; ‘Why does each plant have its name?’
*Value* (n = 30)	Questions inquiring about the importance or role of something, considering individuals’ needs and the capacity of things (and their derivatives) to fulfill them	‘Why is cryptomeria important?’; ‘Why are nature reserves so important?’
*Impact* (n = 19)	Questions focusing on the consequences or effects of an action/event, be it natural or human-made, on the natural environment	‘What would happen if we had not conserved the Azores juniper?’; ‘What would happen if the insects disappeared?’
*Concept* (n = 12)	Questions seeking the definition of a specific concept	‘What does *biodiversity* mean?’; ‘What is a *protected area*?’
*Functionality* (n = 7)	Questions inquiring about the function or utility of something within the ecosystem or for humanity	‘How could the water from the lagoon be used?’; ‘What is the color of plants for?’
*Possibility of knowledge* (n = 4)	Questions asking about the learning potential of a certain action or experience	‘Why is it important to walk on trails?’; ‘Why is it important to be silent to hear nature?’
*Strategic options* (n = 2)	Questions concerning actions and/or processes that participants can implement to mitigate or solve an environmental problem	‘How can we preserve the environment?’; ‘What can I do to help the forest?’

The most frequent type of questions, representing approximately one-third (34%) of the total, are those that require an explanation of a *causality* event. Following closely are questions inquiring about the *nature of things* (21%), and questions about the *value of things* (18%). Only after, appear questions related to the *impacts* of specific events or phenomena (12%) and questions about the definition of concepts (7%). Four questions pertain to the importance of specific actions for the *possibility of knowledge*, and two questions focus on *strategic options* for nature conservation.

When comparing questions of younger and older adolescents, we found no significant statistical differences in the distributions of the questions’ aims (Fig 3A; χ2(7) = 2.768; *p* = .906), unlike what happened when comparing the total valid questions with the TOP-5 ones (Fig 3B; χ2(7) = 21.230; *p* = .003). Although *causality* and *value* have similar frequencies in both groups of questions, we can observe differences in the frequency of questions related to *nature of things*, *impact*, and *concept*, between the two groups.

The *nature of things* loses its significance, representing one-fifth of the total valid questions but less than one-tenth of the TOP-5 questions (7%, n = 4). Similarly, questions related to *concept* also lose importance in the TOP-5 questions group, as participants only selected one question with this aim among the most interesting. In contrast, the *impact* of things or events gains more prominence in the TOP-5 questions group, representing one-third of the questions (33%; n = 18), making it the second most frequent aim in this group of questions.

### RQ3: Which topics are focused on participants’ questions?

**Fig 4:**
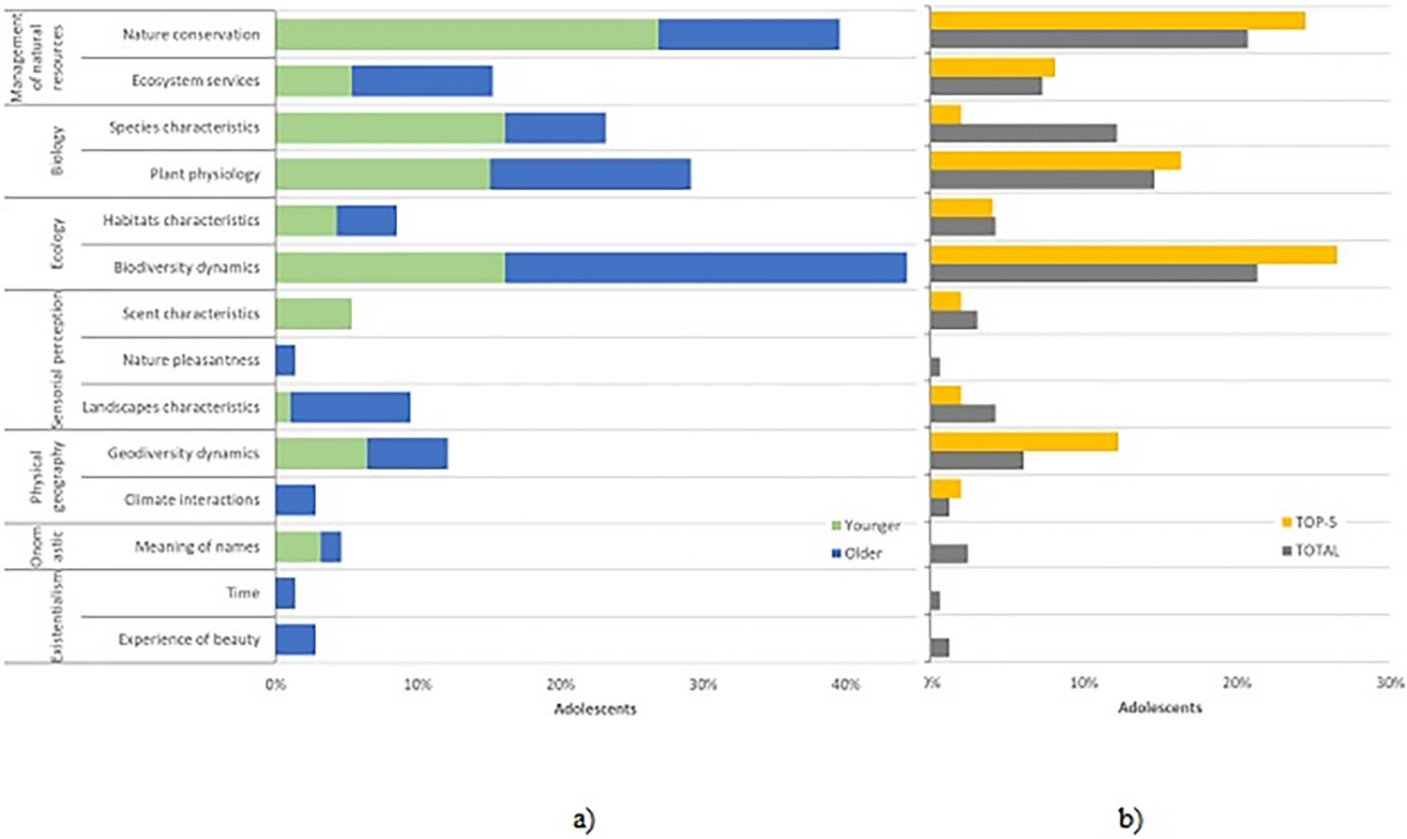
*Topics in focus on participants’ questions about nature* (Younger adolescents: N_Younger_ = 23; Questions_Younger_ = 93; Older adolescents: N_Older_ = 13; Questions_Older_ = 71; Questions: N_Total_ = 164; N_TOP5_ = 55): (a) Comparison between adolescents’ age groups; (b) Comparison between the TOP-5 most interesting and the total valid questions.

We identified 14 topics that the questions focused on (Table 4; Fig 4). Among these, *Biology* (n = 44) and *Ecology* (n = 42) emerged as the most frequent topics, accounting for about half (52.4%) of the questions. In *Ecology*, the prevalent theme was the understanding of *biodiversity dynamics*, representing approximately one-fifth of all the questions (21.3%), including subtopics related to species adaptation, extinction, dispersion, and diversity. On the other hand, questions about habitat characteristics, including specific habitats like forests and lagoons, had a smaller presence (4.3%). Biology encompasses two evenly distributed subcategories: *species characteristics* (12,2%), focusing on descriptive information about species and their distribution, and *plant physiology* (14,6%), inquiring about processes related to the physiology and anatomy of plants.

**Table 4:** Topics and subtopics in focus and respective examples of adolescents’ questions about nature (N_Participants_ = 36; N_Questions_ = 164; n, number of formulated questions in each category).

Topics	Subtopics	Examples
*Management of natural resources*	*Ecosystem services* (n = 12)	‘For what activities could we use the lagoon?’; ‘Why is it important to have vegetation?’; ‘How could the water from the lagoon be used?’
	*Nature conservation* (n = 34)	‘What would happen if we hadn’t conserved the Azores juniper?’; ‘What does it mean to conserve?’; ‘Why is it important to eliminate invasive plants?’
*Biology*	*Plant physiology* (n = 24)	“How is the bark of some trees soft?’; Why is ‘green’ the dominant color in nature?
	*Species characteristics* (n = 20)	‘Describe the Azores juniper.’; ‘Why is there so much Azores juniper in Terceira Island?’
*Ecology*	*Biodiversity dynamics* (n = 35)	‘Why is so reduced the number of birds that we hear?’; ‘What would happen if the diversity of plants were not great?’
	*Habitat characteristics* (n = 7)	‘Do plants ‘choose’ their habitat?’; ‘How are natural forests and lagoons similar?’
*Sensorial perception*	*Landscapes characteristics* (n = 7)	‘Describe what you see on the trail of *Mistérios Negros*.’; ‘Describes the sounds of nature.’
	*Nature pleasantness* (n = 1)	‘Why is nature pleasant?’
	*Scent characteristics* (n = 5)	‘Why are some smells stronger than others?’; ‘Describe the smell of wood.’
*Physical geography*	*Climate interactions* (n = 2)	‘If our island was not so humid would the same species exist?’; ‘Why is it important for the region’s climate to have the characteristics it has?’
	*Geodiversity dynamics* (n = 10)	‘How did the volcanic eruption of *Mistérios Negros* take place?’; ‘What does volcanism mean?’; ‘How are lagoons formed?’
*Onomastic*	*Meaning of names* (n = 4)	‘Why is the *Mistérios Negros* lagoon called *Lagoinha do Vale Fundo*?’; ‘Why does each plant have its name?’
*Existentialism*	*Experience of beauty* (n = 2)	‘What is the role of color in the beauty of nature?’; ‘Describe the beauty of the lagoon.’
	*Time* (n = 1)	‘Why is the time in the forest different from the time in the city?’

The topic *management of natural resources* gathered a similar number of questions (n = 46; 28,0%), which include two subtopics: *nature conservation*, such as protected areas, species conservation, invasive species eradication, and environmental protection in general; *ecosystem services*’ exploring the utility and functionality of habitats and species, including the utility of water. Two topics, *Sensorial perception* (n = 13) and *Physical geography* (n = 12) had much lower representation (less than 10%). The former focused on landscape experiencing through sight, hearing and scent; the latter included geodiversity dynamics associated with *volcanic processes* and *climate interactions*.

Two very distinct categories, with a more limited presence, complete the distribution: Onomastic (2,4%), focusing on the meaning of names of species and places, and Existentialism (1,8%) highlighting the understanding of the subjective *experience of beauty* and *time* in nature.

When comparing younger and older adolescents, the topics of their questions show significant differences (Fig 4A; χ2(13) = 27.91; p < .01). Younger adolescents tend to focus on nature conservation, species characteristics, and plant physiology. In contrast, older adolescents are more inclined to focus on landscape characteristics, biodiversity dynamics, and all the questions related to existentialist topics.

We found no significant statistical differences when comparing the total number of questions with the questions chosen as the TOP-5 (Fig 4; χ2(13) = 19.34; *p* = .113). However, subtopics such as *geodiversity dynamics*, *biodiversity dynamics*, and *nature conservation* are more frequent in the TOP-5 questions while *species characteristics* and *landscape characteristics* are more common in the total questions group.

### RQ4: Why are participants’ questions chosen as the most interesting or relevant to them?

To understand why participants chose specific questions as the most appealing–TOP-5 questions–we asked the 11 groups of participants to justify their choices. From the 34 arguments provided, we identified five types of justifications (Table 5).

**Table 5:** Adolescents’ arguments used to justify their choice of the TOP-5 most interesting questions about nature (N_Participants_ = 33; N_Arguments_ = 34; n, number of formulated arguments in each category).

Arguments	Examples
*To learn something* (n = 15)	‘Because we do not know so we want to know.’; ‘Because we would like to know more about the nature of our island.’
*Due to questions properties* (n = 7)	‘They are the most difficult to answer.’; ‘We have chosen these questions because we find them interesting, and they arouse our curiosity.’
*To reflect* (n = 6)	‘Because we find it interesting to know what would happen if things were not the way they are.’; ‘They are hypothetical.’
*To understand the world* (n = 4)	‘They help to understand the past.’; ‘Because we are interested in what will happen in the future.’
*To justify action* (n = 2)	‘The reason we chose this question is because we all hate insects (e.g. cockroaches).’; ‘We also want to know this because, for example with the question *how can the water from the lagoon be used*?, there may be functions of this water that can bring us some benefits that we still do not know.’

Almost half of the arguments (44%) are related *to learning something*, such as solving a knowledge gap, acquiring new knowledge, or simply because learning is seen as beneficial. About a fifth of the justifications (21%) pertain to the *properties of the questions* themselves, particularly because they aroused curiosity, posed difficult or ’ill-structured problems,’ or prompted *self-reflection* (18%), encouraging exploration of hypothetical scenarios and questioning assumptions. Additionally, around one-tenth of the justifications (12%) revolve around *understanding the world*, either to comprehend the past or predict the future. Only two arguments aim to understand the implications of an action or the utility of something (*justify action*).

## Discussion

### Implications for knowledge on adolescents’ curiosities about nature

This study aimed to examine adolescents’ inherent curiosity about nature as expressed through self-generated questions. By exposing 36 adolescents to a rich natural environment and utilizing the cultural probes technique to encourage their exploration, we collected 164 valid questions about nature, with 137 unique and distinct, all within the course of a single field visit. To the best of our knowledge, this is the first study to employ self-generated questions to comprehend the appeal of nature to adolescents.

As observed by other authors, adolescents tend to perceive nature as a system comprising biotic and abiotic elements with minimal human impact (S1 Table). Our findings indicate that participants find life (e.g. species) more intriguing than abiotic elements (e.g. habitats, volcanic eruptions, climate) (Table 2), similarly to Puhakka & Hakoköngäs [64] study. The predominant subtopics explored by the participants further support this observation: questions concerning biodiversity dynamics, specifically species adaptation, extinction, dispersion, and diversity, were the most popular, accounting for over one-third of the questions chosen as the most interesting. These results are consistent with the findings of Baram-Tsabari and colleagues [65], who analyzed scientific topics within self-generated questions from children and adolescents. Contrary, the aesthetic conceptualizations of nature and the pleasantness of nature were rare and were not considered the most interesting by the adolescents, aligning with the findings of Pointon [29]. Issues related to the interaction between humans and nature appear to be very relevant to these adolescents, not in the recreation or restorative sense found by Tillmann and colleagues [11], but particularly focusing on nature conservation, which echoes the growing concern of eco-anxiety in youth [66]. Additionally, participants displayed a specific interest in botany, with plant physiology ranking as the third most appealing topic for their questions (Table 4), which is most unusual given in the existing plant awareness disparity [67].

Interestingly, while questions related to species characteristics, including plant physiology, decreased in significance in the TOP-5 questions, inquiries about the impacts of actions or events (natural or human-made) on the natural environment emerged as the second most appealing aim.

Even within a natural setting, diverse curiosities could surface, not all related to nature and its immediate concerns, such as human well-being or even the media to share their experiences and observations. However, when considering both the objects and topics of the questions posed by adolescents, we find that the vast majority are focused on nature, whether aiming to understand elements and phenomena, or seeking to comprehend its added value and utility, and/or expressing the need to preserve it. These findings suggest, as expressed by Soga and Gaston [35], that interest in nature is not declining in a homogeneous way and, adolescents may show substantial “depth of understanding of nature”, as proposed by Keith and colleagues ([4], p. 1547) working with young urban Australians.

As expected, considering the studies by Cobern and colleagues [27], younger adolescents exhibit a broader scope of justifications, focused not only on their interests, curiosities, and knowledge gaps, but also on a desire to understand the world and how to contribute to problem-solving. In contrast, older adolescents appear to be more self-centered, prioritizing personal concerns over a focus on learning how to address and resolve broader world issues [23], which could be an interesting hypothesis to explore. However, significant differences between the two age groups were only observed regarding ‘nature conservation’ issues, with the younger adolescents posing more questions related to the protection of the environment as a whole, although this is not observed in the ranking of the TOP-5 questions, where nature conservation ranks second, nearly on par with ecosystem dynamics. The divergence between age-groups somewhat echoes the already signaled *time-out* [20, 35], or *dip* [23], found in various western countries concerning the diminished engagement of adolescents with nature, environment and sustainability [4].

Although this inflexion needs to be fully understood, it may be hypothesized that the developmental tasks faced by adolescents in constructing their identity [68] mediate a temporary dip. Besides, when adolescents try to discover who they are, which values they will adopt and their purpose in life, they critically question values and norms previously taken for granted and move away from learned behaviors practiced in childhood and puberty. All of these may constitute critical distancing strategies necessary for development. Meanwhile, the *shifting baseline syndrome* is occurring, where generational amnesia leads to increasingly lower reference points for what is considered a meaningful interaction with nature [69], normalizing lower levels of exposure and interaction with the natural world [8, 38].

The justification of the most interesting questions lies in propositional knowledge. Approximately two-thirds of the justifications for the TOP-5 questions are driven by a desire *to learn something* or to satisfy curiosity about complex and ill-structured problems [70], that may not be addressed within a typical school setting. Conversely, only a small proportion of adolescents expressed an interest in acquiring procedural knowledge–learning how to act or *justify actions*.

Considering the influence of the ’setting’ on the questions formulated can offer valuable insights into the interplay between context and the emergence of situational interests and/or curiosity. In our study, older adolescents were the sole participants who raised questions related to volcanic processes, after exploring this topic on Station 13. Similarly, younger adolescents showed evident curiosity about lagoon formation following their task on Station 7. While constituting less than 5% of the total questions, these examples underscore the significance of place-based learning in shaping adolescents’ interests and inquiries, as already proposed by Fattorini and colleagues [71]. This also aligns with findings by Mattouk and Talhouk [72], who conducted a content analysis of nature photographs, where adolescents incorporated visions of local culture on their representations of nature. Therefore, it is pertinent to examine the occurrence of questions in more significant categories, such as nature conservation. Among younger adolescents, 20 out of 25 questions related to nature conservation emerged during the activities directly focused on this issue (Stations 4 and 5). In contrast, among older adolescents, only five out of nine questions arose after engaging in activities directly related to conservation (Stations 9 and 13). These examples highlight that the setting undeniably influences the content of situational interests, although the impact is not consistent and requires further investigation to establish a systematic pattern.

Moreover, the disparities observed between the distributions of questions generated *in situ* and those selected as the most interesting (TOP-5) during the post-trail activity offer a unique perspective on the role of the setting. The most frequently asked questions *in situ* did not necessarily make it to the TOP-5, not even among the age group that originally proposed them. The absence of motivational activities and exposure to the nature-rich environment created a distinct context in which the questions, now printed on cards, became the foundation for adolescents’ subsequent discussions and final choices.

### Implications for practice

#### Expanding curiosity and interest in nature

Engaging adolescents in non-formal, question- and place-based learning activities within local nature-rich environments showed that such experiences could appeal to adolescents’ and provide insights into their connection with nature [12, 33]. These activities fostered curiosity and directed attention towards elements that may not have been previously appreciated or valued. Actually, Giusti and colleagues [73] found that education professionals identified “feeling comfortable in natural spaces”, “being curious about nature”, and “caring about nature” as the most indicative abilities of Human-Nature Connection (HNC).To further develop nature curiosity among adolescents, the process requires a thoughtful approach that considers not only the characteristics of the activities and settings but also the readiness of the learners to respond to them [57]. Bentz and O’Brien [74] highlight the potential of transformative learning by using art in empowering youth to engage with climate change, suggesting that educational strategies fostering active participation and reflection can effectively enhance adolescents’ connection to nature. Several researchers have contributed to this area, proposing models and strategies to consider. One such model, developed by Harackiewicz, Smith, and Priniski [45], outlines four types of interventions to promote interest and achieve educational outcomes: (i) structural features, including attention-getting settings; (ii) context personalization, which evokes prior individual interests; (iii) problem-based learning; and (iv) utility value, where the activities are designed to enhance practical purposes. In addition, Renninger, Bachrach, and Hidi [57] identify, among other things, potential triggers for interest, such as affect, autonomy, challenge, technology (computers), novelty, and personal relevance.

Despite the complexity and idiosyncrasy of the process leading to the expansion of HNC, calling upon pre-existing adolescents’ individual curiosity, seems relevant for education and conservation. This is doable when formal education accommodates bottom-up and top-down curricular components. Our study is a case in point: it was possible to unveil many themes that could be addressed during classes. Adolescents justified their choices of TOP-5 questions with the *desire to learn* about issues often perceived as not related to school. Strategies for incorporating meaningfulness into school [45, 75] and approaching students to science and nature include the voices, needs and interests of adolescents in the contents of learning, using approaches such as inquiry-, project-, and problem-based teaching. Besides, our methodological approach, prompting adolescents to question their surroundings in a nature-rich environment, may illustrate inquiry-based teaching, which has positively influenced students’ engagement with STEM [76].

Constructing questions that problematize our perceived reality is not an immediate task and requires nurturing curiosity. For instance, we found that some of the adolescents’ questions (16%) were more strategic than productive [53], in the sense that they did not express a ‘real’ interest in the topic but aimed to complete a specific task to reach the following one. On the other hand, schools tend to deal with simple, well-structured, initiatory problems, which are considerably distant from scientific questioning, mainly dealing with complex, uncertain and ambiguous ill-structured problems [77]. Thus, promoting inquiry-based teaching methodologies seems to be desirable for meaningful learning. Adolescents’ questions may guide teaching objectives and contents, allowing educators to know what interests and does not interest them and what is alien to their interests. For example, our results show that *natural elements* were preponderant in the formulated questions, aiming at their description, explanation and valuation. However, these topics are far from exhausting the knowable universe that nature provides: educational goals focused on contemplation, fruition, impact analysis, procedural knowledge on nature conservation, amongst other topics, can expand cognitive, emotional, and sensorial ways of experiencing nature.

#### Promoting reconnection with nature and conservation responsibility

Research shows that childhood connection with nature can foster hope and resilience in the face of environmental challenges [78]. In this sense, it seems essential to create place-based opportunities in natural rich environments [79], taking the classroom outdoors, into the field. Despite the consensual recognition of its importance by educators and parents [80], safety issues and other barriers prevent children and adolescents’ outdoor time [81]. However, several informal educational movements claim increasing contact with nature in schools (e.g., [82]), and a considerable body of knowledge (cf. review in [30]) may be used to help designing strategies to develop a closer connection to real nature. Notwithstanding, different authors argue that the experience of nature alone may not be enough to foster environmental engagement and responsibility. It is essential to promote active involvement in meaningful pro-environmental actions, allowing adolescents to engage in identifying, critically evaluating, and solving real-world problems [83]. This process should foster autonomy and social support [20], helping them experience success and competence in achieving different goals [84], and/or build resilience to frustration. To enhance a reconnection with nature and increasing the sense of responsibility for its protection, some authors (e.g., [23, 30]) advocate that the practices of environmental education and education for sustainable development need to become closer to approach ESD 2 - “building capacity to think critically about what experts say and to test ideas, exploring the dilemmas and contradictions inherent in sustainable living” ([83], p. 191). This model, conceptualized by Vare and Scott [83], intends to promote the capacity to think critically, test ideas, and discuss and explore dilemmas and contradictions, avoiding a prescriptive teaching approach, typical of the current normative tradition (ESD 1 –“promotion of informed, skilled behaviours and ways of thinking, useful in the short-term where the need is clearly identified and agreed” ([83], p. 191). This prescriptive tradition is disliked by older adolescents [3], who will probably respond negatively to it [23]. The exploration of dilemmas in the school context could also illustrate the ESD 2 framework (e.g., [85, 86]).

## Conclusion

The use of self-generated questions as a research approach in the context of nature’s appeal to adolescents holds immense promise. Our study revealed a wide range of curiosities and/or interests among adolescents, resulting in 137 distinct questions out of a total of 168 formulated about nature. Despite the strong presence of the local bio-geo-physical context in the interests shown by adolescents, these findings align with previous qualitative studies on perspectives about nature, mainly focusing on biotic elements and causal aspects of natural phenomena and processes. Although the interaction between humans and nature play an important role on adolescents’ questions, emphasizing nature conservation, arguments related to procedural knowledge are very rare, suggesting detachment from action for or in nature, indicating a preference for understanding nature rather than actively engaging with it.

This study represents one of the few empirical approaches to understand adolescents’ curiosity about nature using a natural-rich environment, in a collaborative design, to capture adolescents’ voices. The descriptive-interpretative approach justified the convenience sample of two scouts’ groups, but a maximum variation purposive sampling could be useful to test further our findings in the future.

Formulating questions about experiences in non-familiar environments is challenging. To help guide adolescents’ information-seeking about nature, we employed question tokens, which proved to be effective [53]. However, the use of tokens might have constrained participants’ ability to generate questions, potentially limiting the diversity of ideas expressed. Nevertheless, some participants chose not to use any tokens and freely produced their questions.

Although younger and older adolescents explored the same nature trail at the same time, the stations and tasks proposed to each group were not the same. However, the general structure and purpose of the tasks were quite similar, mobilizing the same multiple intelligences, maintaining the ecosystem’s scale and inviting adolescents to walk along the same landscape continuum. Such similarity allowed comparability between groups.

Throughout this work, we realized that there is still much to be understood regarding the engagement of young people with nature and actively involve them in its conservation. Further research is needed to understand (i) the prevalence and relevance of the adolescents’ dip in a rich-natural environment such as the Azores–is it found with nature conservation, with nature in general, or even, more broadly, with environmental and sustainability issues?; (ii) the length and process of emergence from the dip–when and how do adolescents reconnect with nature? Which factors mediate the end of the time-out? Which experiences are crucial in this process? What is the role of the local context in this process?; (iii) the involvement in nature conservation–is the onus of sustainability perceived as a personal matter or as a matter that others should take care of? Why?. A multiple-generation cohort longitudinal study would help clarify these questions.

## Supporting information

S1 Table Synthesis of studies conducted with children and adolescents on perspectives, connection, and ways of experiencing nature. We conducted a scoping review (Grant and Booth, 2009) of research focusing on children and adolescents’ perspectives, connections, and experiences with nature, in a tabular form. The aim was to characterize the goals, methods, and scope of the existing evidence. The search process was guided by keywords related with the aims (theme) of the studies. Quantitative studies that assessed nature connectedness or examined its predictors were excluded, as our priority was to explore children’s representations and experiences on their own terms. The search was conducted in English, and no temporal or geographical restrictions were applied.
(DOCX)
