# Supplementary material for: Discovering the allure of forests: Exploring adolescent queries in nature-rich environments
Source: PLoS One. 2025 Jan 16;20(1):e0312955. doi: 10.1371/journal.pone.0312955 (PMC11737762; doi:10.1371/journal.pone.0312955)
Supplement: S1 Table — We conducted a scoping review (Grant and Booth, 2009) of research focusing on children and adolescents’ perspectives, connections, and experiences with nature, in a tabular form. The aim was to characterize the goals, methods, and scope of the existing evidence. The search process was guided by keywords related with the aims (theme) of the studies. Quantitative studies that assessed nature connectedness or examined its predictors were excluded, as our priority was to explore children’s representations and experiences on their own terms. The search was conducted in English, and no temporal or geographical restrictions were applied. (DOCX) [file pone.0312955.s001.docx]

**Table S1** Synthesis of studies conducted with children and adolescents on perspectives, connection, and ways of experiencing nature. We conducted a scoping review (Grant and Booth, 2009)^[[1]](#footnote-1)^ of research focusing on children and adolescents’ perspectives, connections, and experiences with nature, in a tabular form. The aim was to characterize the goals, methods, and scope of the existing evidence. The search process was guided by keywords related with the aims (theme) of the studies. Quantitative studies that assessed nature connectedness or examined its predictors were excluded, as our priority was to explore children’s representations and experiences on their own terms. The search was conducted in English, and no temporal or geographical restrictions were applied.

| **Study** | **Participants**  (N; age; place) | **Data collection techniques** | **Data analysis** | **Findings** |  |
| --- | --- | --- | --- | --- | --- |
| **Theme: How do children and adolescents conceptualize nature?** | | | | | |
| Wals, 1994 | N=32; 11-13 years old; Detroit, USA | Interviews | Interviews transcription; creation of a framework with 'family-related' patterns conducting to an emergence of categories. | Five features emerged: nature is flowers, animals, trees and it's alive; real nature is pure, peaceful, pristine, and not human-made; nature is freedom; nature is solitude; nature is self-supporting, wild and spontaneous. Participants usually shared more than one theme. |  |
| Cobern et al., 1999 | N=16; 14-15 years old; Arizona, USA. | Individual interview; concept maps. | Content analysis | Five co-existing worldviews inspire adolescent's perspectives of nature: scientific [less prevalent than expected], religious, aesthetic, conservationist, and utilitarian; individual perspectives result from an idiosyncratic synthesis among some or all of them. |  |
| Kong et al., 1999 | N=52; 12-20 years old; Singapore. | Focus groups | Discussions were taped and transcribed; then interpretations and organizing initial themes and topics. | Two oppositions emerged: Nature as `familiar' vs. nature as `other'; Nature as unpredictable and dangerous vs. nature as safe and fun. Participants endorse urban development priorities over nature conservation. |  |
| Aaron and Witt, 2011 | N=50; 9-11 years old; Houston, USA. | Semi-structured interview, drawing activity and description of the drawings | Researchers utilized Atlas.ti.5.2 Scientific Software to organize, code, and generate themes from the collected interviews using the constant comparative method. | Three themes emerged: natural elements; outdoors/outside; nature is not in the city. |  |
| Pointon, 2014 | N=384; 13-14 years old; England. | Open-ended questions about their understanding of and relationship with ‘nature’. | Inductive thematic analysis | Predominant anthropocentric worldview, excluding humans, and emphasizing science and utility; few participants held an ecocentric worldview, including aesthetic themes and an intrinsic value relationship; about a third of participants held a human-related view, where humans are seen as both a part of and yet separate from nature, recognizing the ‘otherness’, integrity, and the intrinsic value of nature. Concepts shaped by urban/rural location, gender and school type. |  |
| Collado et al., 2016 | N=832; 6-12 years old; Spain. | Free evocations with the stimulous word ‘nature’. | Content analysis | Four themes emerged: natural and non-natural elements [more prevalent]; human-nature relationship; emotional experience of nature; actions in natural settings. The type of daily experience with nature influences individual conceptions. |  |
| Keith et al., 2022 | N=1,269; 8-14 years old; Sydney, Australia. | Open-ended questions about perceptions of nature, written in student’s own words | Inductive thematic analysis, focusing on both common and unique patterns | Most students viewed nature positively, often associating it with beauty and peace. A smaller portion of the students expressed ambivalence or negative views, with some describing nature as "scary" or "ugly". |  |
| Sjöblom and Wolff, 2017 | N=678; >14 years old to early university students; Finland. | Stratified sampling with a descriptive questionnaire followed by semi-structured interviews. | Content analysis assisted by software QSR Nvivo 8.  Thematic analysis.  Validation through research triangulation | Six complex forms of valuing nature: material (resources); aesthetic; recreational; biodiversity (due to ethical motives); future (sustainability); holistic (environmental interdependence). The majority of the participants expressed three to four values. |  |
| Tillmann et al., 2019 | N=84; 9-14 years old; Ontario, Canada. | Focus groups | Revision of transcripts and assigning text to the three components (definitions, experiences, benefits) using NVIVO Pro (Version 11). Thematic analysis to identify patterns within the data set. Combination of deductive and inductive coding to develop a categorical organizing framework for the analysis. | There is a tension when defining nature between considering nature as a sum of many parts (a descriptive theme centered around activities, natural elements, and locations, often excluding humans), or as 'a whole community' (focusing on the links between natural elements), and, on the other hand, seeing nature through a specific natural element, such as a classroom plant. |  |
| Zamora et al., 2021 | N=994; 14-24 years old; MyVoice interactive SMS platform | Open-ended questions | Thematic analysis, and descriptive statistics of answers posted in an interactive SMS platform. | Five themes emerged: natural elements (trees/woods; animals/bugs); outdoors; green-spaces (gardens); sensations (peaceful, beauty); environment (Earth, not man-made). |  |
| **Theme: How do children and adolescents connect and experience nature?** | | | | | |
| Wals, 1994 | N=32; 11-13 years old; Detroit, USA | Interviews | Interviews transcription; creation of a framework with 'family-related' patterns conducting to an emergence of categories. | Eight experiences of nature emerged: entertainment, a background to activities, a challenging place, a reflection of the past, a place to reflect, a place for learning, a threatening place, and a threatened place. Nature is perceived as familiar and safe, but also challenging, unpredictable, and prompting frightening and threatening experiences. |  |
| Kong et al., 1999 | N=52; 12-20 years old; Singapore. | Focus groups | Discussions were taped and transcribed; then interpretations, and organizing initial themes and topics. | Nature [for young urban Singaporeans] is seen as challenging, unpredictable, and prompting frightening and threatening experiences. |  |
| Kahn et al., 2002 | N= no information; 12-14 years old; Houston, USA; Amazonia, Brazil; Lisbon, Portugal | Interviews, photo questionnaires, and surveys | Both qualitative and quantitative methods: factor analysis, content analysis, and inductive thematic analysis. | Differences in how adolescents perceive and engage with nature, based on their urban or rural context, age group, and prior experience with natural settings. |  |
| Owens and McKinnon, 2009 | N=58; 13-20 years old; California, USA | Open-ended survey focused youths’ views of their community, not including questions about use or feelings towards nature, retrieving 1300 photos | The survey responses were transcribed verbatim into a qualitative analysis software program (Nvivo 7). Photographs were attached to the corresponding written responses and provided a useful means for verifying the written response. | Three themes emerged relating to youths’ activities: recreation, restoration, and socializing. The occurrence of nature is widespread in the place’s adolescents use, and is highly valued; tasks associated with social behavior and self-awareness are evident. |  |
| Aaron and Witt, 2011 | N=50; 9-11 years old; Houston, USA. | Semi-structured interview | Researchers utilized Atlas.ti.5.2 Scientific Software to organize, code, and generate themes from the collected interviews using the constant comparative method. | Positive feelings about nature (freedom, fun, tranquility; 'a good feeling') usually prevailed over negative ones (fear) or failing to notice it.  Nature is valued by all children, but some of them are unable to justify why; others underlined physical and mental health benefits, the need of protection and a desire to learn from it. Preferences regarding nature: being outside and active (freedom, adventure) are preferred by the majority; being inside is associated with fear and discomfort of nature, and technological appeal; some students don't get to choose. |  |
| Linzmayer and Halpenny, 2014 | N=5; 6-10 years old; Alberta, Canada. | Creative expressive visual methods (drawings, analysis of expression of child in pictures) and repeated (5) semi-structured interviews | Inductive thematic analysis | All children demonstrated a potential for empathy and a concern for certain parts of nature. Two main themes were found: 'being attracted to nature' (we take care of nature; nature takes care of us) and 'a repulsion from nature' (we need protection from nature; nature needs protection from us - family and children may act as gatekeepers). |  |
| Adams and Savahl, 2015 | N=32; 13-14 years old; South Africa | Focus groups | Inductive thematic analysis | Five experiences of nature emerged: nature as a threatened place; culture of inconsideration towards nature; nature as external to the self; nature as the dangerous other; and intrinsic care for nature. |  |
| Mattouk and Talhouk, 2017 | N=77; 7-16 years old, rural villages in Lebanon | Photovoice to capture children perceptions of nature.  They also wrote narratives to explain their images | Inductive thematic analysis performed on both photographs and written narratives | Perception of nature closely tied to agriculture (backyards, orchards - domesticated nature) in opposition to natural landscapes and wild nature.  Family, particularly grandparents, are essential in shaping positive views of nature.  Nature was also seen symbolically, reflecting the participants’ inner emotional states. |  |
| Tillmann et al., 2019 | N=84; 9-14 years old; Ontario, Canada | Focus groups | Revision of transcripts and assigning text to the three components (definitions, experiences, benefits) using NVIVO Pro (Version 11). Thematic analysis to identify patterns within the data set. Combination of deductive and inductive coding to develop a categorical organizing framework for the analysis. | Children enumerated specific ways of engaging with nature (e.g., hunting, fishing, swimming, playing outside, climbing, exploring, building forts, running, walking, and games) more than addressing the resonance of nature experience. Very few sports and other structured activities were mentioned; children favored free play or made-up activities. Adolescents primarily associated their experience of nature with the contexts or places they use for recreational, learning or reflection activities, such as playing, hiking, or camping. |  |
| Hickman et al., 2020 | N=no information; Results are discussed between children and adolescents | Interviews and questionnaires | Inductive thematic analysis | Identified that eco-anxiety is increasingly prevalent among young people, with feelings of helplessness and distress commonly reported. |  |
| Zamora et al., 2021 | N=994; 14-24 years old; MyVoice interactive SMS platform | Open-ended questions | Inductive thematic analysis, and descriptive statistics of answers posted in an interactive SMS platform. | The participants reported feeling physically and mentally better when spending time in nature (calm, reduced anxiety, more active) and these positive feelings prevailed over negative ones (7%) (isolation). Most youth wanted to spend more time in nature, but some mentioned barriers (i.e., busy schedules, built environment, and COVID-19) impeding them from doing so. |  |
| Cerv et al.,2024 | N=108; 11-13 years old; Austria, Vienna. | Children’s took photos representing what nature means to them, selected one, and wrote a short description.  The "Inclusion of Nature in Self" (INS) scale measured their connectedness to nature, | Inductive thematic analysis supported by MAXQDA software.  INS scores were used to investigate the relationship between nature connectedness and perceptions. | Most photos (50.9%) were taken in urban areas, followed by natural areas (35.2%), and at home (13.9%).  Students with higher connection to nature took more photos in natural settings.  Many students expressed positive emotions toward nature in their descriptions. |  |

1. Grant MJ, Booth A (2009) A typology of reviews: an analysis of 14 review types and associated methodologies. Health Information and Libraries Journal 26:91-108. [↑](#footnote-ref-1)
